# Points of contention: Qualitative research identifying where researchers and research ethics committees disagree about consent waivers for secondary research with tissue and data

**DOI:** 10.1371/journal.pone.0235618

**Published:** 2020-08-05

**Authors:** Angela Ballantyne, Andrew Moore, Karen Bartholomew, Nic Aagaard

**Affiliations:** 1 Department of Primary Health Care and General Practice and the Bioethics Centre, University of Otago, Wellington, New Zealand; 2 Philosophy, University of Otago, Dunedin, New Zealand; 3 Waitematā and Auckland District Health Boards, Auckland, New Zealand; 4 Ethics, Health System Improvement and Innovation, Ministry of Health, Wellington, New Zealand; University of Texas Southwestern Medical Center at Dallas, UNITED STATES

## Abstract

**Background:**

This is a multi-method, in-depth, three part qualitative study exploring the regulation and practice of secondary research with tissue and data in a high-income country. We explore and compare the perspectives of researchers, research ethics committees (RECs) and other relevant professionals (e.g. pathologists and clinicians). We focus on points of contention because they demonstrate misalignment between the expectations, values and assumptions of these stakeholders.

**Methods:**

This is a multi-method study using observational research, focus groups and interviews with 42 participants (conducted 2016–2017) and analyzed using thematic analysis.

**Results:**

Results are arranged under the following themes: consent; balancing the social value of the research with consent requirements; and harm. Our findings demonstrate different perspectives on the review process, styles of ethical reasoning and issues of concern. First, researchers and RECs disagreed about whether the cost of re-consenting patients satisfied the criterion of impracticability for consent waivers. Second, most researchers were skeptical that secondary research with already collected tissue and data could harm patients. Researchers often pointed to the harm arising from a failure to use existing material for research. RECs were concerned about the potential for secondary research to stigmatize communities. Third, researchers adopted a more consequentialist approach to decision-making, including some willingness to trade off the benefit of the research against the cost of getting consent; whereas RECs were more deontological and typically considered research benefit only after it had been established that re-consent was impractical.

**Conclusion:**

This research highlights ways in which RECs and researchers may be talking past each other, resulting in confusion and frustration. These finding provide a platform for realignment of the expectations of RECs and researchers, which could contribute to making research ethics review more effective.

## Introduction

Since the 1980s demand for tissue for research has grown dramatically [1], and this demand is expected to continue as genomic, post genomic and precision medicine research activities expand [2]. Demand for health data is also increasing to fuel research in artificial intelligence, big data analytics and learning health care systems (which support the continuous generation of knowledge to inform the provision of care). Data-intensive medical research challenges the traditional 'consent or anonymise approach' approach to ethical data regulation [3]. Health and genomic data are now being shared, re-used, linked and analysed on an unprecedented scale, often outside the parameters for which the data was originally collected.

Many countries, including New Zealand, have set national strategic priorities to build a knowledge economy, and to intentionally harness data-driven technologies, science and innovation to drive economic growth [4]. Clinicians are encouraged to support medical research by generating research questions, facilitating consent in the clinic for the future use of biological specimens and data for research, and supporting research translation back into the clinic [5, 6]. This may lead to relatively inexperienced clinician-researchers proposing to undertake research with data or samples they encounter in their clinical roles.

However, the regulatory structure for secondary use of health information and tissue is complex and difficult to navigate, even for experienced health researchers. Such research may fall under multiple regulatory instruments including legislation, regulation or policy relating to privacy, data protection, human tissue use and human subjects’ research. This regulatory ecosystem aims to support socially valuable health research and innovation, while protecting privacy and public trust [7]. The evolving and increasingly interconnected nature of scientific research points to a complex web of relationships requiring trust, stewardship and responsible governance [8]. Hoeyer asks “what can be done to make biobanks into trustworthy institutions of long-term social durability?”[9] This is a complicated balance that requires the regulatory system to simultaneously:

1. protect the existing public trust in health and research institutions;
2. establish a social and cultural licence for the sharing of tissue and data for research;
3. facilitate access to research material (tissue and data);
4. protect patients’ privacy and other legal rights; and
5. prevent or minimise harm arising from data and tissue misuse or poorly designed or communicated research.

Research Ethics Committees (RECs) and Institutional Review Boards (IRBs) have an important role in this regulatory process. Many jurisdictions and international guidance documents allow an IRB/REC to waive the consent requirement for secondary use research, in cases where gaining consent would be impractical or would impede the scientific validity of the study; where the study addresses important health questions; and poses minimal risk of harm to data subjects [10–13]. Guidelines typically require that *all* three criteria are satisfied in order to grant a consent waiver. The new EU General Data Protection Regulation gives member states freedom in determining whether patient consent is necessary for secondary use of health data for medical research [14]. If RECs/IRBs decline to grant a waiver of consent, researchers must abandon the research or re-consent all patients and participants whose tissue or data is included in the dataset. Previous research has shown RECs feel a heavy burden of responsibility to protect patients’ and publics’ interests when patient consent is not sought [15, 16].

Regulation of health data is broadly considered by researchers to be a barrier to conducting health research [15, 17]. Even in the context of more traditional clinical research, RECs come under criticism from researchers on the grounds that they are secretive [18, 19], inconsistent [20, 21], slow [22], and the ethics review process is unduly burdensome [23, 24]. There is a recently emerging literature on how RECs function and make decisions [15, 25–30]. Our research adds to this growing literature and is particularly interesting because it compares the opinions of researchers and RECs, illuminating different perspectives on the review process, styles of ethical reasoning and issues of concern in relation to secondary research. In highlighting these different approaches to ethical reasoning, we hope to provide a constructive platform for greater understanding between researches and RECs.

In this paper we provide a comprehensive picture of the points of contention between New Zealand researchers, RECs and other stakeholders regarding secondary use research. We report on qualitative research involving all four national-level RECs in New Zealand (called Health and Disability Ethics Committees or HDECs), interviews with researchers and supplementary interviews with surgeons and pathologists. In this paper we focus on philosophical and to some extent legal understandings of consent for research; but we note that consent is sociologically constructed and that our framing only sheds light on some aspects of the consent process [31]. This research focuses on comparing the perspectives of REC members, researchers and other professionals We do not directly investigate patients’ or research participants’ views; and we acknowledge that consent, while a primary focus of this paper, is alone an inadequate solution to participants’ concerns about the use of tissue and data in secondary research [9, 32].

In New Zealand, the National Ethics Advisory Committee–Kāhui Matatika o te Motu (NEAC) issues the National Ethical Standards for Health and Disability Research and Quality Improvement (the Standards) in line with its statutory functions as described in the New Zealand Public Health and Disability Act 2000. HDECs check that research protocols meet the ethical standards set out by NEAC and they must act consistently with New Zealand law (including for example the New Zealand Bill of Rights Act 1990, the Health and Disability Commissioner Act 1994, the Human Tissue Act 2008 and the Privacy Act 1993). These requirements likely shape and frame HDEC views about the ethical issues discussed in this research.

Our study is a unique multi-dimensional, in-depth view of the regulation and practice of secondary research with tissue and data in New Zealand. We go beyond the existing critiques of RECs, to demonstrate the different ways researchers and RECs analyse ethical questions about tissue and data research. This study is important because it demonstrates where researchers and RECs are talking past each other.

## Methods

This paper draws on data from three related qualitative studies conducted in New Zealand in 2016–2017. The overall aim was to understand different stakeholders’ perspectives on the ethical issues and regulatory processes relating to secondary research with clinical tissue or data in New Zealand. Further details of the methodology (including the research protocols and interview guides) are provided in S1 Appendix.

Study 1: Focus groups and interviews with members of all four New Zealand national-level research ethics committees—HDECs. In addition, we conducted three observational sessions with the HDECs and multiple discussions with the Ministry of Health HDEC Secretariat and the HDEC Chairs. This study used a participatory observation approach (AB was a long-serving member of one of the HDECs) and thematic analysis. The full methodology is described in a previous paper [15]. Here we present results that were not included in the initial publication. HDEC Chairs have provided feedback and contributed to data interpretation for this manuscript. This study was approved by the Human Health Ethics Committee at the University of Otago H16/090.

Study 2: Semi-structured interviews with thirteen researchers who had applied for approval for a consent waiver for secondary research through the national HDECs process. Participants included both researchers whose applications for consent waivers had been approved and those that had been declined. This study used purposive sampling and thematic analysis. This study was approved by the Human Ethics Committee at the University of Otago 16.028.

Study 3: Semi-structured interviews with five stakeholders (three surgeons and two pathologists). The third study was a small spin-off pilot study that gathered additional data on specific themes emerging from the earlier two studies. This study used purposive sampling and snowballing; and thematic analysis. This study was approved by the Human Health Ethics Committee at the University of Otago H16/034.

Focus groups were conducted in person and interviews were conducted in person or via the phone. Written or oral consent was obtained (and recorded) from all participants. All focus groups and interviews were audio recorded and transcribed by a professional transcriber. Nvivo was used to support data coding and data analysis. All focus groups and interviews were conducted by AB and used semi-structured interview templates, which are attached in the S1 Appendix. AB and AM conducted the coding and data analysis for study 1. AB and AM developed the coding scheme and coded the transcripts for studies 2 and 3. In addition KB and AG read select sections of the transcripts for study 2 and 3 and contributed to data analysis and interpretation.

Transcription and data analysis were conducted iteratively. Saturation was defined as the point at which no new themes were emerging [24]. We reached data saturation for studies 1 and 2 but not for study 3, which was a pilot study and did not aim to reach saturation.

Summary results and draft papers were presented orally and/or in writing to the MOH secretariat and to the Chairs of the HDECs (who had the option of sharing these results with their respective committees). This process helped refine and validate data interpretation. The varied expertise of the research team, combined with the process of stakeholder engagement, provided a depth of perspective to support, challenge and deepen the data analysis and interpretation [33].

## Results

The data summarised here are drawn from three complementary sets of interviews and focus groups involving a total of 42 participants.

- #1–4 refer to the four HDEC focus groups, including 22 members (members asked not to be identified as individuals)
- #5 and #6 are follow-up interviews with individual HDEC members
- #7–19 refer to interviews with researchers who had applied to HDECs for a consent waiver to access tissue or clinical data without patient consent
- #20–24 refer to interviews with other relevant stakeholders (surgeons and pathologists)

New Zealand is a small country of 4.8 million people and the professionals we spoke to often had experience in multiple roles—as clinicians, researchers, ethics reviewers, setting up tissue banks, management roles. In general the interviews were broad-ranging and addressed multiple perspectives on the issue of secondary use research. The cohort included researchers and clinicians with experience in both the public and private research sectors.

Here we present the most interesting and important points of contention between RECs, researchers and other stakeholders on ethical issues in secondary research. Due to this focus we do not present on other themes present in the data—for example, there was discussion, in all three participant groups, about the difference (technical, ethical, sociological or cultural) between tissue and data. This is perhaps unsurprising given New Zealand has a research framework called Te Mata Ira which provides culturally informed guidelines for biobanking and genomic research that take account of Māori (the indigenous people of New Zealand) views, concerns and expectations about the sharing and use of tissue and DNA in research [34] However in this paper we focus on points of contention because they demonstrate misalignment between the expectations, values and assumptions of RECs and researchers. Results are arranged under the following themes: consent; balancing the social value of the research with consent requirements; and harm.

While we focus on points of contention in this paper, we also note that approximately half the researchers we interviewed thought the REC review process was overall constructive.

### Consent

Some researchers were surprised that they would need ethics approval to access biological samples or data for research.

“A lot of these patients are deceased, so we thought that we could probably just go to the labs, get the tissue, and go with it. But um, what we found out pretty quickly was no, that can’t be done.”

(#9)

#### Re-consenting patients

In cases where the HDEC required researchers to re-consent patients, many researchers thought this was unnecessary, prohibitively expensive or caused harm and anxiety to patients. In no case had the REC review process convinced researchers that re-consenting patients was necessary.

“…this is always voluntary research that we do it just in our own time, and so we never have a lot of time to do it, and to feel like you’re you know, [re-consenting patients] really felt so unnecessary… And that was pretty upsetting for me… it put me off wanting to do that sort of depth of research study again, because it just, it felt so unnecessary and so misunderstood from the committee… its’ yeah, an incredibly frustrating process.”

(#14)

“And we actually got to a point where the [REC] gave me provisional approval… but, what we had to do to get to that point of provisional approval, my boss has just said, this is completely impractical and you just can’t do it. But [REC] kept going on about why couldn’t I employ a nurse to get consent. “

(#10)

Researchers were also concerned about the potential for the re-consenting process to cause distress to patients or relatives.

“I felt that it was potentially harmful [distressing] actually… I wasn’t worried about the work entailed for us.”

(#11)

#### It’s too expensive to re-consent patients

An important point of contention that emerged between researchers and RECs was the question of whether the *cost* of re-consenting patients satisfied the criterion of impracticability. Cost was not specifically mentioned in the national NEAC Guidelines for Observational Studies [26] in place at the time of the research (the Guidelines have since been updated) (see Table 1).

**Table 1:** NEAC guidelines for observational studies.

Section 6.43 Access to identified or potentially identifiable data for research (without consent) may be justifiable when:
a) obtaining consent would cause either:
• unnecessary anxiety
• prejudice the scientific value of the study; or
• it is impossible in practice due to the quantity or age of the records; and
b) there would be no disadvantage to the participants or their relatives or to any collectivities involved; and
c) the public interest in the study outweighs the public interest in privacy.

Note that the national guidelines have since been updated: the National Ethical Standards for Health and Disability Research and Quality Improvement were published in December 2019.

RECs were mainly of a view that cost alone did not satisfy the impracticality condition.

“I think when you start putting cost over people’s rights, you are actually on a slippery slope… So, I wouldn’t necessarily be looking at the cost, that’s not my problem.”

(#1)

“I mean [researchers have] had some doozy excuses over the years, can’t be bothered, it’s too much cost…”

(#4)

More specifically, RECs expressed some concern that researchers relying on cost considerations may not have sufficient resources to adequately perform research.

“[if they]…don’t have the resources to be able to [re-consent]…‥you’d have to question; is that researcher going to be adequately competent to be able to do the study, if they’re not …capable of meeting ethical obligations.”

(#6)

An exception here was REC members who were also researchers. This sub-group were more sympathetic to the cost implications of research.

“If it was a study that was hugely beneficial to society as a whole, and would answer a really important question that you couldn’t answer any other way, I would certainly consider [cost].”

(#5)

Researchers however typically thought that cost was a relevant consideration for assessing impracticality. “…the added costs of that would have been prohibitive. … so in every sense, we couldn’t have done it [if consent was required].” (#18)

“…this was a project without any resource…and it would have required me to go and get consent… We would need additional funding for that.”

(#15)

#### Original consent

We heard from RECs that some researchers argued consent for secondary research use of data and samples had been obtained during the standard clinical consent process at the point of collection (either during informed consent for surgery or when a patient enrolled with a health service). Our interviews with researchers confirmed this perspective. RECs expressed skepticism that this would qualify as valid informed consent, even broad consent.

“I mentioned [to the REC] that all patients have signed consent, and that consent form actually says ‘I agree to my tissue being used for future research, provided ethical approval is obtained’. And I quoted that, and I got shot down at the committee, and told that that only applied to audits.”

(#12)

### Balancing the social value of the research with consent requirements

RECs and researchers had different perceptions of the relationship between the potential benefit of the study and the need to get consent. Researchers typically weighed these two features against each other; so that if the research was especially beneficial or important, and a consent process would be costly and challenging to conduct, this would justify not getting consent.

“… [we should consider]‥; the greater good, the greater good of the community; … So I think it is a balance.”

(#17)

“…you think of the potential benefit of you know the women [in the target group], and then weigh it up again with what harm would you be doing any of the women who have already been through the system and been treated. Well it was overwhelming, you know, in our view…we had a moral obligation to go ahead and do this study without individual consent.”

(#12)

However RECs typically dealt with these two issues sequentially. There was a clear (though not unanimous) consensus amongst RECs that researchers must first justify why getting consent would be impractical; only then, would the REC consider whether the public interest in the research was sufficient to grant a waiver.

“You can’t just say; this is public good because it’s about diabetes and fat people in South Auckland, so we won’t even bother getting consent. You know, the first thing is; get consent.”

(#4)

“I suppose the first question is; is it possible to get consent at all? And if so, how much of a problem would that be?”

(#1)

This difference in approach was a major source of misunderstanding between researchers and RECs. RECs expressed frustration with researchers’ attempts to justify consent waivers primarily on the grounds of public benefit.

“[the research] was great, and it would have saved people, everything was good about the research, but you just can’t justify getting people’s tissue without consent…because under NZ law, you need to try and get consent…”

(#4)

“…this research may have a great deal of social benefit, but if trust is destroyed when it gets published, [its] a short term benefit for long term loss; it’s important that people trust their clinicians.”

(#1)

Researchers thought that RECs were often undervaluing the benefits of the research.

“[patients] would be horrified to think that stuff was being thrown away that might be useful.”

(#11)

“And all these slides are sitting in off-site storage in boxes and they are effectively lost for future generations because we struggle so much to get permission to look at them… And it really is dormant material now to a significant extent.”

(#12)

“….[using] tissue banks or/and data warehouses for capturing information on these hard to capture areas or populations and disadvantaged communities is, I think, common sense.”

(#2)

Interestingly, individual members of RECs who were also health researchers put greater emphasis on the potential benefits of the research in their reasoning.

“I’m a researcher and I would say; I quite like this. This has public good, and meets my criteria.”

(#4)

“The benefit seems very solid. Very immediate, very immediate.”

(#5)

### Harm

Most researchers were skeptical that secondary research with already collected tissue and data could harm patients. In relation to concerns about privacy and confidentiality, clinician researchers suggested the risks in research were minimal, especially compared to the magnitude of the risk of a privacy breach in normal clinical care.

“No, there was only benefits really, …[the] people having access to identifying data, where all those who would have normally had access through normal health systems. And so there was no particular risk.”

(#18)

“[privacy and confidentiality] weren’t an issue [in the research]… I think god, I see so much stuff every day in my normal day to day job, I mean honestly.”

(#10)

Several researchers expressed the idea that the primary harm came from *failure* to use clinical tissue and data to improve care.

“Particular samples that we have stored are an incredible reservoir of information…Where the harm comes in, is if we don’t have access to these samples because of regulations being so tight that they must be discarded as soon as the diagnostic testing has been done.”

(#8)

#### Stigma

RECs identified the potential for data and tissue research to stigmatize sections of the community as a major concern associated with secondary research. In this context, stigma meant that the research results could be portrayed in a way that made unflattering or discriminatory generalizations about sections of the population.

“I think that the stigmatisation issue in a country like New Zealand, is huge. “

(#5)

The warrior gene case was referred to as an example by one REC (#1). This was a case in New Zealand where a genetic association study was reported as showing that Māori carry a gene linked to a range of anti-social behaviours including violence [35].

However, most researchers were perplexed by RECs questions about stigma and several mentioned that this had been a point of contention during REC review.

“…if you’ve got [condition x], you’ve got [condition x], and I just think it’s better to know than to be worried about stigmatising people, because if we identified that there is more among Māori, then we can start talking … about some specific targeted screening programme… I think it’s all Orwellian trying to legislate [against stigmatization], and I think it’s crazy.”

(#10)

“I was asked at the ethics committee meeting how could we ensure that we weren’t stigmatising these women? And the reality is, that if you’re going to get that politically correct, how are you ever going to solve the [health] problem?”

(#12)

A minority of researchers could see the *potential* for research to stigmatize but no researchers thought that stigmatization was a real risk in their specific research project.

“[the REC] asked about whether …this work would stigmatise a person or a population, and quite specifically Māori … And that’s fair enough, because I think we need to think about things like that.”

(#17)

#### Evidence and authority

One point of contention related to who was best placed to identify risks and predict research harms—RECs or researchers? REC members, clinicians and researchers all brought a particular perspective based on their personal experience, and often they drew on multiple perspectives to bolster their authority (e.g. I am a clinician researcher and I have also been a patient). Some researchers implied that all these supposed risks considered by the HDEC were essentially hypothetical, whereas the researchers *know* what data/tissue research actually involves and therefore *their* judgement should carry more weight.

“I think, because I know what’s being done with [the tissue], [the risk] it’s minimal. “

(#9)

“….scientists do have the attitude…of ‘trust me, I know what I’m doing, and I’m doing it in good faith, and let’s get on with it’”

(#8)

“…one of our [REC] members will say; she consents people all the time, she’ll say; nobody ever cares about this. But it’s our job to care about it for the one person that might.”

(#4)

Researchers often thought HDECs were being overly cautious; whereas HDEC members worried that researchers lacked the broad experience necessary to foresee certain research risks.

#### Limitations

First, some of the research was conducted several years ago. This is a multiple dimensional study involving several stakeholder groups. The participatory co-design approach was time consuming because it involved significant input and consultation with the HDEC Chairs and the MOH in New Zealand, throughout all stages of the research. As a result of this process we able to build trust and conduct focus groups with all national level RECs in New Zealand. This rich qualitative data is a valuable contribution to the growing literature on the REC decision-making.

Second, it is possible that researchers were more likely to agree to participate in this study if they were dissatisfied with the REC process and/or their application had been rejected. The MOH does not collect data on the annual number of applications for consent waivers nor the proportion that are approved, so we are not able to benchmark the rate of waiver approvals amongst our participants against an external standard. However, participants included researchers whose applications had been approved and rejected and they expressed appreciation for and frustration with the ethics review process.

## Discussion

Here we focus on three points of ethical contention emerging from the research. We focus on points of contentions because these are primary sources of dissatisfaction and stress for both researchers and REC members and highlighting these issues provides a constructive platform for better mutual understanding and alignment in the future. The exiting literature suggests researchers experience significant dissatisfaction with ethics review and our novel findings help illustrate issues where RECs and researchers seem to be talking past one another. First, there is debate about whether the costs of re-consenting patients should satisfy the ‘impracticality’ requirement. Second, our results suggest that researchers adopt a more consequentialists approach, and will trade off the benefit of the research against the cost of getting consent; whereas RECs are more deontological, and only consider public benefit once it has been established that re-consenting is impractical. Third, our results demonstrate the struggle for epistemic authority between different members of the REC and between researchers and RECs.

### Does prohibitive cost satisfy the impracticality criterion?

A clear point of contention between researchers and RECs was whether the cost of re-consenting was a relevant consideration. The NZ NEAC Guidelines (at the time) stated the condition for waiver of consent as being that consent would be “impossible in practice due to the quantity or age of the records” [36]. By comparison, a review of the US Cardiovascular Register explicitly cites financial cost and methodological reasons as justification for not gaining patient consent to use health data [37]. Whether cost is relevant will depend on whether the “quantity” of records is an absolute or relative measure. RECs in New Zealand seem to favor an absolute interpretation of quantity, but none specified what this amount would be (e.g. impractically would apply to any study using data from more than 500 patients). Researchers favored a relative interpretation, where the practically of re-consenting is measured relative to the project resources available. For an un-funded study, even re-contacting 50 patients for consent may be prohibitive. The relative/absolute distinction will have a significant impact on how much secondary research can be conducted, and who can participate in this research (clinicians undertaking researcher versus established well-funded research teams); and potentially what sorts of research questions are asked, if we think that clinicians have specific or different insight into unresolved medical questions. Researchers are in a difficult position if their institutions or funders will not adequately support the costs of re-consenting participants where necessary. Our results suggest that RECs may be using the issue of resourcing/funding to wean out poorly-funded and possibly less experienced researchers, about whom RECs seem especially concerned. RECs see themselves as having an acute protection function when granting waivers of consent [15]; but this cautious approach may exclude potentially valuable research.

In defense of NZ RECs, satisfying the impractically ground simply on the basis of cost relative to resources would risk establishing perverse incentives in research. Unfunded researchers would easily meet the impracticality condition interpreted in that way. Research that has successfully undergone competitive grant funding and peer-review, which represents an independent check on the social value of the research, would have greater difficulty meeting this criterion. But there is merit to researchers’ claim that the resources available for re-consenting are relevant to the question of feasibility. Resource constraints are widely accepted to be relevant to medical decision making.

The international consensus is that cost is relevant to the impractically of re-consenting. A comparative review of legal and ethical frameworks governing the secondary use of data for research purposes in the United States, Canada, the United Kingdom, Australia and France concluded that an important point of *consensus* between these five jurisdictions is that they all recognize that a waiver of consent could be ethically justified when consent is impracticable “e.g., when costs are prohibitive” [38]. The revised 2016 international research guidance from the Council for International Organizations of Medical Sciences (CIOMS) states: “The most common justification for using records or materials collected in the past without consent is that it would be impracticable or prohibitively expensive to locate the persons whose materials or records are to be examined” [39].

We endorse the position advocated in CIOMS which requires a global assessment of the risks and potential benefits of the study: “The aggregate risks of all research interventions or procedures in a study must be considered appropriate in light of the potential individual benefits to participants and the scientific social value of the research” [39]. The merits of re-consenting patients should be assessed relative to feasibility (including the project resources), the social value of the proposed research, and the degree of risk to patients.

### Ethical reasoning

A notable pattern of ethical reasoning has emerged from the data—researchers tend to use consequentialist reasoning and will weigh the public benefit of the research against the cost of getting consent; whereas RECs tended towards deontological reasoning, which is represented by their sequential analysis of consent, followed by public benefit.

Consequentialist reasoning emphasises the outcome of the actions that are being assessed, whereas deontological reasoning focuses instead on the nature of those actions themselves and of the intentions these express. Consequentialists seek to maximise the overall value of the consequences (e.g. in terms of welfare, public benefit), while deontologists seek to act in ways that in themselves are ethically required (e.g. respecting rights) and not in ways that in themselves are ethically prohibited (e.g. using others as mere means).

Debates between consequentialists and deontologists can quickly become pointless because the debaters are using different ethical frameworks, and resolving disputes between the frameworks themselves is a complex and difficult task. Our results demonstrate a process of RECs and researchers often talking past each other. This finding could explain a significant amount of the frustration expressed by both researchers and RECs in this study, and in previous research. Interestingly, when we reported the preliminary research results to RECs there was interest in this finding, and we had the impression that it helped REC members to better understand some of the clashes between RECs and researchers. A dissenting narrative was observed where REC members who were also researchers were more sympathetic to consequentialist reasoning. It will be important to see if these findings are confirmed in future empirical research with RECs and researchers.

That RECs adopt more a deontological approach (than researchers) is perhaps a predictable finding, given that RECs are regulatory committees tasked with applying rules and principles. Some philosophers have argued that RECs are essentially administrative committees whose job it is to check research protocols for consistency with the applicable codes of research conduct [40]. Conversely, Schaefer defends the scope of RECs to engage in free-flowing ethical analysis [41]. We do not take a position here on whether the REC job is to review research proposals for their consistency with the applicable policy standards for research conduct, or is instead to review for their ethical acceptability. If RECs really are doing open-ended ethical reasoning, we might expect to see a greater balance between consequentialist and deontological reasoning.

We do not take a position here on whether consequentialist or deontological approaches are preferable. Elements of both style of reasoning will be relevant as part of the RECs role is to protect research subjects rights (deontological); and as we argue above a global assessment of the relative merits and burdens of the research is also necessary (consequentialist). This pluralistic approach is widespread in research ethics and is explicitly articulated in the 1979 Belmont Report [42].

### Epistemic authority

Social epistemology is the study of knowledge as a social practice, including the dynamics of testimony, authority, communal analyses of evidence, epistemic agency and social conceptions of objectivity.[43]. Both members of RECs and researchers asserted their privileged standpoint and their superior ability to judge the potential harms of research. This can be interpreted as an attempt to claim epistemic authority, thereby centering their perspective in the process of knowledge production.

Epistemic authority is especially important in relation to all *non-consensual* research (not just secondary use research) where researchers, REC members and in some cases additional institutional review boards (in New Zealand this may include Māori research review) or patient representatives try to predict the perspectives or non-consenting research subjects. This process involves attempting to identify the interests and preferences of future research subjects, weighing competing interests on behalf of research subjects, and trying to ameliorate risks in ways that would be acceptable to research subjects. During this process, different stakeholders may assert their epistemic authority to judge on behalf of research subjects. We found many instances of this, particularly in relation to whether stigma is a ‘real’ risk in secondary research.

Hamersley argues that sound ethical judgment requires contextual knowledge [44]. Both REC members and researchers drew on experiences outside their nominated role to bolster their claim of expert knowledge. Prior research has found that in situations where REC members have no professional experience, they will draw on personal experience during ethical decision making [23, 45]. In this study, REC members drew on their experience outside of the committee—as clinicians, researchers and patients, as well as involvement with specific communities (e.g. Pasifika or Māori) or conversations with family members or other members of the public. Researchers referred to their experience as clinicians to demonstrate their knowledge of patients’ concerns (or lack of them) regarding secondary research. Clinician researchers were more attuned to the potential harm arising from failing to undertake socially valuable research [36, 46, 47]; whereas this potential harm seemed less visible to RECs.

Running across all three themes was the particular challenge of clinicians with limited research experience, aiming to conduct research with samples or data already in their possession. This cohort was more likely to lack specific research funding, be ‘isolated’ researchers and to be conducting research on top of their current clinical workload. We did not ask specifically about the level of experience of researchers. This impression was based on how researchers described their previous work experience and their proposed research project. These researchers expressed the most intense frustration and dissatisfaction with the research ethics review process. It is plausible that these were senior clinicians, with an expectation of epistemic authority in their clinical field, who felt patronized or vilified by RECs for their lack of familiarity with the research review process and expected ethical standards. This cohort of researchers also seemed to cause the most anxiety for RECs. This finding has implications for countries adopting national research strategies that seek to embed research into clinical practice, for example in the form of learning healthcare systems [48]. Research governance is a complex framework and those new to research need to be appropriately supported to navigate this process.

Epistemic authority is a valuable lens for understanding conflict between researchers and RECs. Questions of experience, expertise and authority arise in areas where RECs are called on to assess novel research designs and/or where there is limited policy guidance [49–51]. A previous survey of New Zealand health researchers found researchers perceived deficiencies in the expertise on ethics committees, particularly their expertise to judge the potential harms of research [52]; and our research demonstrates RECs questioning both the research and ethical competence of some researchers. Dove and Garattini identified a lack of REC expertise in data-intensive research, specifically data-linkage, and RECs efforts to align this research with traditional notions of “specific” consent [53]. Klitzman found that in cases where IRBs perceived a lack of relevant local knowledge and were worried about uncertainty and risk, they adopt a precautionary approach and may end up being overly paternalistic [54]. In our study, researchers perceived that RECs were overly cautious and attributed this to a lack of REC expertise and contextual knowledge.

## Conclusion

Data sharing is an essential driver of learning health systems, artificial intelligence and precision medicine. Internationally RECs provide the core regulatory oversight for sharing clinical data and tissue. Our study is innovative in that it provides a multi-dimensional view of the ethics review process for secondary research. REC processes are notoriously frustrating, and often confusing, for researchers. Our research provides detailed insight into the reasoning of RECs and is an important contribution to transparency in the data ecosystem.

Here we have focused on identifying clash points between researchers, RECs and other stakeholders. Points of contention are important because they demonstrate misalignment between the expectation, values and assumption of RECs and researchers. Our research shows that these clash points are sources of stress, anxiety, and conflict among the parties.

There were three key insights that emerged from the research. First there was disagreement as to the role of ‘cost’ in satisfying the ‘impracticality’ criterion for consent waivers. In general researchers thought that cost was relevant; whereas RECs resisted the influence of cost. Second, we identified a difference in approach to ethical reasoning. Researchers took a more consequentialists approach and RECs adopted a primarily deontological approach. Finally our research demonstrated the struggle for epistemic authority between researchers and RECs; with both asserting their superior positions to represent the interests of patients and populations and to accurately identify the benefits and (especially) the risks of research. For example, most researchers were skeptical that secondary research with already collected tissue and data could harm patients, and they were particularly perplexed by RECs concern about the potential for research to stigmatize data subjects.

We hope these insights may provide a platform for mutual understanding and re-alignment of the perspectives of RECs and researchers. We conclude by offering several suggestions to support re-alignment. System leaders should clarify the role of RECs and IRBs with respect to whether committees are essentially administrative (and therefore may give effect to their ethical perspectives here only insofar as those perspectives express and apply the duly established and applicable policy standards) or have scope for free-form ethical analysis and are able to reject protocols on the grounds of their own ethical judgment [40, 41]. As demand for health data and tissue continues to grow, it may also be worth establishing specialist RECs with expertise in data science and biobanks [55]. Research ethics guidelines need to be regularly updated to address novel research methodologies. Revised NEAC guidelines have been released in New Zealand subsequent to this research and they offer greater clarity on the ethical standards for tissue and data research. It is imperative for regulators to clarify whether resource limitations are sufficient grounds for not re-consenting patients. Inexperienced researchers should receive training and support to help them translate a burgeoning research hypothesis into an ethical, code-consistent, safe research protocol. It would be valuable to provide forums for greater researcher and REC interaction, for example joint-training days when new ethical guidance or policy is released. These semi-formal environments could provide a forum for relationship building between RECs and researchers and joint discussion of emerging research methods and the ethical issues arising.

## Supporting information

S1 Appendix Research protocols and interview guides. (PDF)

S1 File (PDF)
