# Supplementary material for: Points of contention: Qualitative research identifying where researchers and research ethics committees disagree about consent waivers for secondary research with tissue and data
Source: PLoS One. 2020 Aug 5;15(8):e0235618. doi: 10.1371/journal.pone.0235618 (PMC7406047; doi:10.1371/journal.pone.0235618)
Supplement: S1 Appendix — (PDF) [file pone.0235618.s001.pdf]

S1 APPENDIX: Research protocols and interview guides

1. Approving access to clinical tissue and data without patient consent – the views of HDECs in NZ

Researchers: Angela Ballantyne and Andrew Moore

Research aims: The regulatory structure in New Zealand (and many other jurisdictions) allows ethics committees to grant waivers to consent for secondary use research with clinical tissue or data. In judging when to grant these waivers, ethics committees are charged with weighing the social good of the research with the public interest in privacy. Ethics committees therefore have unique experience in working through these ethical issues. The purpose of the research is to better understand how committees weigh the potential social good of the research against the patients' interests in autonomy and privacy. Using vignettes and a semi structured interview approach, in a focus group and individual interview setting, we will map and articulate the process of ethical analysis that committees engage in on this topic. This study was approved by Human Health Ethics Committee at the University of Otago H16/090.

The research methodology for this component to the research is presented in a prior publication.[1]

Focus groups lasted between 32 and 64 minutes; interviews lasted 31 and 55 minutes. The participants were 19 women and 5 men, roughly matching the gender distribution of the members of these HDECs.

**Areas of expertise of HDEC members who participated in the research**	
observational studies	3
intervention studies	7
intervention/observational studies	1
consumer/community perspectives	8
ethical and moral reasoning	2
health/disability service provision	2
Law	1

Interview template:

Presentation of two vignettes involving secondary use research

1. What ethical issues would the committee considered when assessing these sorts of applications?

---

[1] Angela Ballantyne & Andrew Moore (2018) Data and tissue research without patient consent: A qualitative study of the views of research ethics committees in New Zealand, AJOB Empirical Bioethics, 9:3, 143-153,DOI: 10.1080/23294515.2018.1518938

2. What elements inform your judgments about the potential public good of the research? /How do you interpret public good in the context of this sort of research?
3. What elements inform your judgments about the public interest in privacy? /How do you interpret privacy in the context of this sort of research?
4. How do you balance the interest in public good and the public interests in privacy?
5. What cultural factors are relevant to the review of these studies?
    a. cultural views about the value and use of tissue;
    b. trust in researchers and/or governance bodies who manage biobanks and registries;
    c. risk of stigma to populations;
    d. studies that may have high public good for particular groups
6. Is there any information you are missing that you feel would help you make a judgment about authorizing access to patient data and tissue samples without consent?
7. Are applications that seek to access clinical data or tissue harder to assess than other observational studies? If so, what features make these hard?

2. Use of clinical data and biological sample for research in New Zealand: understanding the pathways.

Researchers: Angela Ballantyne and Andrew Moore

Aims: The project aims to understand how researchers in New Zealand navigate and perceive the regulatory restrictions and processes around accessing clinical tissue and data for research without consent. The research will give us a better insight into the pathways that researchers use to access clinical material for research. Researchers are balancing financial constraints, desire to progress medical research and ethical and professional obligations to patients. We want to better understand their reasoning and their views on whether the current regulatory system strikes the right balance between public good and patient's autonomy, or whether the system is overly restrictive in providing access to clinical material or too liberal. This study was approved by the Human Ethics Committee at the University of Otago 16.028.

Interview template:

- Describe the research study for which you sought an HDEC waiver
- Describe the social value of this research
- What, if any, do you think were the risks to communities or patients of proceeding without consent? Prompts: privacy, harm, stigmatisation, commercialization, cultural issues?
- Did HDEC grant the requested waiver/ approve the study?
- Did you agree with the HDEC decision, in particular the emphasis the committee placed on social benefit, privacy, consent, risk, feasibility?
- Some have suggested that medical RECs don't have sufficient expertise in relation to data or tissue studies, what is your view on this?
- Did you have any governance processes in place to allow for community input into the research?
- Would it have been feasible to do the study, or a component of it, if patient consent had been required?

- Are there other avenues you have used to access clinical data or tissue for research (eg 'research' clauses on surgical consent forms, evaluation or audit mechanisms)?
- Are there any other research studies that you have been involved with that relate to these themes that you would like to discuss?

Recruitment: Purposive sampling was used to select researchers who had submitted an application to a national level research ethics committee (called HDECs in New Zealand) for approval for a consent waiver to access clinical data or tissue samples for secondary research. HDEC minutes are public and the AB reviewed the minutes of the four HDECs to identify potential participants. 30 researchers were invited via email to participate in the interview study in 2016 and 2017; 14 did not respond; 1 indicated that her study did not fit the criteria; 1 provided comments via email but did not participate in an interview and one agreed to participate but we could not find a suitable time for an interview.

Participants: 13 researchers participated in interviews in person or via the phone in late 2016. Nine participants were male and four were female. Oral consent was obtained and recorded. All interviews were audio recorded transcribed by a professional transcriber.

3. Surgical consent forms in New Zealand – understanding their role in the use of clinical tissue for research.

Researchers: Angela Ballantyne, Karen Bartholomew, Andrew Moore, Richman Wee, Nic Aagaard.

Aims: The research will give us a better insight into the pathways that researchers use to access clinical material for research within the evolving structure of biobank establishment in New Zealand. Evidence presented in the earlier two studies suggested significant variation amongst researchers and the protocols at different hospitals and pathology laboratories regarding releasing and authorizing clinical material for research without explicit patient consent (Note: 'waiver' of consent is possible (under specific parameters) through an application to the Health and Disability Ethics Committee, therefore we are considering research that is within the current regulatory framework). This research may have ethical, regulatory and legal implications as it pertains to the Human Tissue Act 2008 and the Health Information Privacy Code 2014. This study was approved by the Human Health Ethics Committee at the University of Otago H16/034.

Interview template:

- Does the surgical consent form at your institution list research as a possible use of clinical samples?
- If so: When was this introduced? Do you know anything about the motivation for including researcher on surgical consent forms (who made the decision, was there consultation or discussion and who was involved, what was the intended purpose)?
- How is this clause currently interpreted and used?
- Does it allow researchers access to clinical samples for research, i.e. is it considered to represent patient consent to research? Is additional oversight/approval required for research – eg from a local or national ethics committee, or an institutional research committee? What sorts of research does this consent authorise (eg low risk, only with anonymised samples)? Who can access clinical samples for research (only local institutional investigators, or also university researchers, or researchers from other DHBs?)
- Other avenues for accessing clinical information.

- Have you ever applied to an HDEC for permission to access clinical or archival tissue for research without having to get explicit patient consent? Do you have any comments on that process? How did the HDEC make the decision to approve or decline your request?
- Have you ever applied to a university ethics committee (or other non-HDEC) for permission to access clinical or archival tissue for research without having to get explicit patient consent? Do you have any comments on that process? How did the ethics committees make the decision to approve or decline your request?
- Future Unspecified Research: In your research have you ever used a FUR form in order to provide on-going access to the research samples and data for future research?
- If yes, in your mind how does FUR differ from access to clinical databases for research
- Which is more valuable, are they suited for different purposes?
- What does it cost (financially or in terms of other resources) to manage FUR databanks/datasets over time?
- If you had easier access to clinical tissue and medical records would you be less inclined to get FUR?
- In your mind how do research clauses on surgical consent forms compare to FUR consent forms (are they similar, different, equivalent)?

Recruitment: In addition to research ethics approval (at Human Health Ethics Committee at the University of Otago, application H16/034) we also need to apply for locality at approval at the District Health Boards where we intended to recruit. Some DHBS also required approval by the local Māori research committee. We received locality approval at four DHBs (Waitamata, Auckland, Capital and Coast, and Hutt). We aimed to recruit surgeons, hospital managers, and/or clinical laboratory pathologists in New Zealand. We used purposive sampling to identify experts and then snowballing. Potential participants were approached via email.

Participants: We interviewed five participants in 2016 and 2017. Oral consent was obtained and recorded. All interviews were audio recorded transcribed by a professional transcriber. One interview was conducted by a research assistant and four were conducted by AB. We interviewed two (pathologists with experience running hospital laboratories and with tissue banks) and three surgeons. This cohort included two females and three males.
