# Supplementary material for: Points of contention: Qualitative research identifying where researchers and research ethics committees disagree about consent waivers for secondary research with tissue and data
Source: PLoS One. 2020 Aug 5;15(8):e0235618. doi: 10.1371/journal.pone.0235618 (PMC7406047; doi:10.1371/journal.pone.0235618)
Supplement: S1 File — (PDF) [file pone.0235618.s002.pdf]

AJOB Empirical Bioethics

ISSN: 2329-4515 (Print) 2329-4523 (Online) Journal homepage: https://www.tandfonline.com/loi/uabr21

Data and tissue research without patient consent: A qualitative study of the views of research ethics committees in New Zealand

Angela Ballantyne & Andrew Moore

To cite this article: Angela Ballantyne & Andrew Moore (2018) Data and tissue research without patient consent: A qualitative study of the views of research ethics committees in New Zealand, AJOB Empirical Bioethics, 9:3, 143-153, DOI: 10.1080/23294515.2018.1518938

To link to this article: https://doi.org/10.1080/23294515.2018.1518938

Published online: 08 Nov 2018.

Submit your article to this journal

Article views: 117

View related articles

View Crossmark data

Full Terms & Conditions of access and use can be found at
https://www.tandfonline.com/action/journalInformation?journalCode=uabr21

AJOB EMPIRICAL BIOETHICS
2018, VOL. 9, NO. 3, 143–153
https://doi.org/10.1080/23294515.2018.1518938

Taylor & Francis
Taylor & Francis Group

Check for updates

Data and tissue research without patient consent: A qualitative study of the views of research ethics committees in New Zealand

Angela Ballantyne[a] and Andrew Moore[b]

[a]Department of Primary Health Care and General Practice and the Bioethics Centre, University of Otago, New Zealand; [b]Department of Philosophy, University of Otago, New Zealand

ABSTRACT
Purpose: Secondary use of clinical tissue and data is an increasingly important platform for health research. Many jurisdictions allow research ethics committees (RECs) or institutional review boards (IRBs) the flexibility to waive the requirement for patient consent for secondary research. But most RECs/IRBs conduct their meetings "behind closed doors" and their decision-making processes are opaque to researchers and academics. The purpose of this study was to assess how New Zealand RECs weigh the potentially competing goals of enabling research and protecting patients' rights. **Methods:** We used a participatory observation approach involving observation sessions (3), focus groups (4), and individual interviews (2) with members of the national-level health and disability ethics committees (HDECs) in 2016. **Results:** Twenty-four HDEC members participated (75% participation rate). Participants described the core ethical issues as consent, public benefit, and potential harms (to both collectives and individuals). Participants felt the weight of responsibility in waiving patients' right to consent. Time pressure and a lack of specificity in the guidelines resulted in increased anxiety and stress. Participants' comments demonstrate multiple different methods for defining and assessing public benefit. **Conclusion:** IRB/REC members have rich experience of moral reasoning regarding research ethics, especially in areas where the official guidance is underdeveloped. Their insights can contribute to the academic literature and suggest improvements in the review process and in ethical regulation and guidelines.

KEYWORDS
Research ethics; qualitative research; research ethics committees; focus groups; consent

Secondary use of clinical tissue and data is an increasingly important platform for health research. As a result, the governance and management of these resources comprise a major focus of current debate in research ethics, public health, and bioethics. There is potentially high public interest in allowing access to biological samples and health data for research into genomics, precision medicine, and comparative effectiveness studies of existing health treatments. Conversely, this research can present harms to collectives (populations, groups, and families) and individuals. Getting the correct balance between facilitating socially valuable research, minimizing potential harms, and recognizing the rights of patients is challenging. Ethical debate has focused on determining the appropriate standards of patient consent; determining the social value of the research; and protecting privacy and confidentiality. These challenges have inspired recent revisions of the research ethics guidance for secondary use of data and tissue from the World Medical Association (WMA 2016), Council for International Organizations of Medical Sciences (CIOMS 2016), and the U.S. Department of Health and Human Services (Menikoff, Kaneshiro, and Pritchard 2017), among others.

Consent can come in various forms: specific, implied, broad, blanket, presumed (opt-out), meta-consent, and dynamic consent. Some consent models—such as dynamic consent (Kaye et al. 2015) and meta-consent (Ploug and Holm 2016)—have been specifically developed in response to the challenges of future secondary use of clinical tissue and data.

Many jurisdictions allow research ethics committees (RECs) or institutional review boards (IRBs) to waive the requirement to gain specific patient consent for the use of clinical data or clinical biological specimens for research, where consent would be impractical or would impede the scientific integrity of the research,

CONTACT Angela Ballantyne angela.ballantyne@otago.ac.nz University of Otago, Wellington 23 Mein St, Newtown, Wellington 6242, New Zealand.

Color versions of one or more of the figures in the article can be found online at www.tandfonline.com/uabr.
© 2018 Taylor & Francis Group, LLC

and where there is high social value in the research. RECs/IRBs therefore have unique experience in trying to balance the social value of research and patients' rights. This study explored the experiences of national-level RECs in New Zealand. The primary aim was to understand when RECs were prepared to authorize research without consent. The research questions focused on (1) which ethical issues the committees considered relevant to approving waivers of consent; (2) how the committees defined public good/ social value; and (3) the decision-making process committees undertake to reach consensus.

Unlocking the expertise of ethics committees is a notoriously difficult task. Most RECs/IRBs conduct their meetings "behind closed doors" (Stark 2012), and their decision-making processes may seem opaque to researchers and academics. The ethics review process has been widely criticized by researchers for being overly burdensome (Stark 2012), secretive (Borgerson 2014; Hammersley 2010), inconsistent (Caplan 1984; Goldman and Katz 1982), and slow (Smith et al. 2004). In response, there has been a recent proliferation of empirical studies investigating the decision-making processes of RECs/IRBs (Klitzman 2013; Wenner 2016; Trace and Kolstoe 2017).

New Zealand research ethics committees may be more open than those in other countries. New Zealand consistently ranks amongst the least corrupt countries in the world on the Transparency International Corruption Perceptions Index (TI-CPI), and this relates to high levels of transparency and accountability in the public sector (Transparency International 2017). The national-level REC system consists of four health and disability ethics committees (HDECs) that meet monthly. (Committees are named according to their region: Southern [STH], Central [CEN], Northern A [NTA], and Northern B [NTB].) HDEC minutes are published on the Ministry of Health (MOH) website and the meetings are open to the public (unless a researcher requests that an application be heard in confidence) (MOH 2014). In addition, there are multiple institutional ethics committees and (at the time of data collection) one private ethics committee. Figure 1 shows the flowchart for determining whether a study requires HDEC level review—basically, any interventional or observational study involving patients, human tissue, or health information that is above minimal risk requires HDEC review.

Under New Zealand (NZ) policy, only two models of consent are acceptable for health research: (1) specific consent, where participants are fully informed (as per the standards outlined in the HDC Code of Rights) and (2) consent to future unspecified research (this is a type of broad or blanket consent to future uses of tissue or data). In addition, some types of research (e.g., with tissue, data, or adults unable to consent) can be approved by an ethics committee in the absence of consent. There is limited instruction available to NZ RECs (including HDECs) for determining the ethical grounds for allowing access to data or biological samples for research without consent. The Privacy Act provides the general framework for promoting and protecting individual privacy in New Zealand (The Privacy Act 1993). It applies to both the private and public sectors and establishes principles with respect to the collection, use, disclosure of, and access to information relating to individuals. The Privacy Act establishes the role of Privacy Commissioner to investigate complaints. The Health Information Privacy Code 1994 is a Code of Practice issued by the Privacy Commissioner under section 46 of the Privacy Act and gives extra protection to health information. The Health Information Privacy Code permits the disclosure of health information where it "is to be used for research purposes (for which approval by an ethics committee, if required, has been given) and will not be published in a form that could reasonably be expected to identify the individual concerned" (Rule 11(c)(iii)) (Health Information Privacy Code 1994). However, the Code does not explain when ethics committees should grant such approval.

The Guidelines for Observational Studies[1] specify the grounds upon which a REC can grant access to identifiable health information:

> 6.43 Access to identified or potentially identifiable data for research (without consent) may be justifiable when:

a. obtaining consent would cause either:
   - unnecessary anxiety
   - prejudice the scientific value of the study; or
   - it is impossible in practice due to the quantity or age of the records; and
b. there would be no disadvantage to the participants or their relatives or to any collectivities involved; and
c. the public interest in the study outweighs the public interest in privacy. (National Ethics Advisory Committee (NEAC) 2012)

Two enactments empower RECs to approve access to tissue for research without consent, but do not

[1]At the time of writing, these guidelines were undergoing review.

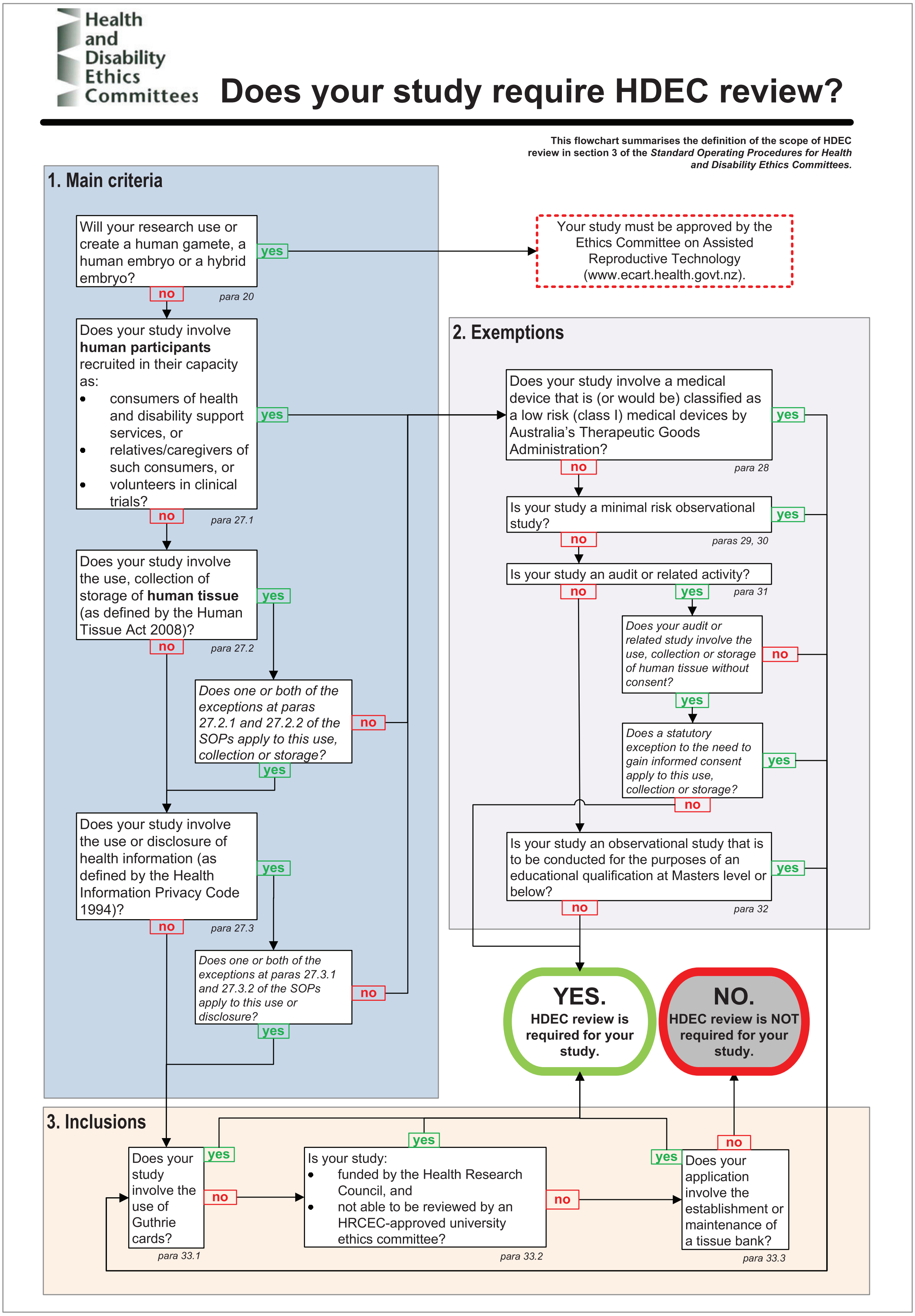

Figure 1. Flowchart to determine whether research requires review by a national level HDEC in New Zealand (2016).

provide additional criteria for determining when RECs should do so. The New Zealand Code of Health and Disability Services Consumers' Rights 1996 provides that:

> Right 7(10) No body part or bodily substance removed or obtained in the course of a health care procedure may be stored, preserved, or used otherwise than:

a. with the informed consent of the consumer; or
b. for the purposes of research that has received the approval of an ethics committee. (HDC Code 1996)

The Human Tissue Act 2008 states that informed consent is not required for collection or use of human tissue for any of the following purposes:

> Section 20 (e) the carrying out … of research that has received the approval of an ethics committee (even though the ethics committee knew that informed consent had not been, and would not be, obtained for the research). (The Human Tissue Act 2008)

Secondary research is challenging because of increasing demand for access to data and tissue among diverse stakeholders, increasing complexity of linkage between datasets, little consensus in the literature regarding when consent is necessary, and in many jurisdictions, including New Zealand, sparse guidance for committees. There is a growing body of empirical evidence showing general, but conditional, public support for data and genomic linkage projects. Public support depends on: trust in the organizations conducting research (Willison et al. 2003); the confidentiality and/or anonymity of the data (Davidson et al. 2013; Xafis 2015); and the use of the data for socially beneficial purposes (Trinidad et al. 2012; Ipsos MORI 2014). The public is more reluctant to allow commercial players access to data (Grant et al. 2013).

The results of this study add to the literature in two ways. First, they enrich the research ethics debate about access to biological samples and data without patient consent, by demonstrating how ethics committees in New Zealand weigh the competing goals of enabling research and protecting patients' rights. Second, the results provide a rare insight into the decision-making processes of IRBs/RECs on a particularly difficult and challenging topic.

Methods

Data collection relied on a participatory observation approach: a method in which the researcher is embedded in the context (Kawulich 2005). Participatory observation is suited to situations where the topic area is sensitive or complex, and where in order to adequately understand the data, the researcher needs to be familiar with the nuance of the context and the issues under investigation. Under participant observation methodology the goal is not for the researcher to remain objective, but for the researcher to be immersed in the community where the research is happening. At the time of data collection, Angela Ballantyne had been a long-serving member of the CEN HDEC. The research design involved collaboration with the chairs of the four national HDECs (Chairs) and with the Ministry of Health HDEC secretariat (MOH secretariat). The MOH secretariat and Chairs contributed to the design of the research method, by, for example,

- Proposing focus groups instead of individual interviews, as this better reflected the group processes of the committees.
- Contributing to the development of the interview template.
- Requesting the use of vignette cases.
- Suggesting observation sessions in addition to the focus group sessions.

Data collection consisted of observations of committee meetings, focus groups, interviews, and ongoing discussions with the Chairs and the MOH secretariat. The focus groups were semistructured and were based around two vignette cases (fictional, but based on real applications submitted to the HDECs) that were distributed to the participants prior to the focus group. The vignettes presented applications to use (1) health information and (2) biological samples for research without patient consent. The vignettes were intentionally concise and ambiguous, in order to force the participants to articulate what further information they would consider relevant to assessing the research. Focus groups were iterative, and issues raised in early focus groups were presented to subsequent focus groups for comment. HDEC members who were not able to attend the focus groups were offered the chance to do an individual interview. Focus groups and interviews were audio-recorded and transcribed by a professional transcriber. Each transcript was returned to the focus group, or individual participant, for comment and amendment. This research was approved by the Human Health Ethics Committee at the University of Otago (H16/090).

NVivo was used to support the coding and analysis of the field notes, focus-group transcripts, and interview transcripts. AB coded the transcripts and took primary responsibility for data analysis and drafting of papers. Co-investigator AM assisted with study design,

question development, transcript reading, development of the coding tree, and the data analysis and interpretation. Respondents are not identified individually as the focus-group design was intended to capture the views of the committee as a group. Quotations from the focus groups are therefore referenced to a committee.

Preliminary results were presented orally and in writing to the Chairs and the MOH secretariat, who contributed to data interpretation. A draft of this article was distributed to the Chairs and MOH secretariat for comment prior to submission, but the final decision on the article's content rested with the authors.

Results

Data collection occurred in late 2016, and data analysis and presentation of results to the participants occurred in 2017. AB observed a meeting of the NTB, NTA, and STH committees and collected field notes (a formal observation was not conducted with the committee that the author serves on). AB then conducted a focus group with each of the four committees, in each case prior to the beginning of a monthly committee meeting. In addition, AB conducted two individual interviews with members. One interview was with a member who was not able to attend the focus group and one was with a member who arrived only in time for the last 5 minutes of the focus group; this participant has been recorded as an interview participant and not counted as a focus-group participant for the demographic information that follows. Focus groups lasted between 32 and 64 minutes; interviews lasted between 31 and 55 minutes.

AB has not counted herself as a participant. Each HDEC has eight members, giving a total of 31 potential participants (excluding the author). Twenty-four members participated in the research (22 members in focus groups, and two interviews), resulting in a 75% participation rate. The participants were 19 women and 5 men, roughly matching the gender distribution of the members of these HDECs (26 women and 5 men). Table 1 shows the primary expertise category of participants (as listed on the HDEC website).

Table 1. Areas of expertise of HDEC members who participated in the research.

Observational studies	3
Intervention studies	7
Intervention/observational studies	1
Consumer/community perspectives	8
Ethical and moral reasoning	2
Health/disability service provision	2
Law	1

Three major themes emerged: (1) ethical issues (consent, public benefit and potential harm); (2) appropriate standards for judging the ethical issues (actual public views, reference populations, and the hypothetical reasonable person); and (3) HDECs' burden of responsibility.

Ethical issues

Participants described the core ethical issues as consent, public benefit, and potential harms (to both collectives and individuals).

Consent

Participants consistently emphasized the primacy of patient consent. The discussion primarily focused on the three authorization options available under NZ regulations—specific consent, broad consent for future use, and REC/IRB authorization in the absence of patient consent.

Participants expressed a clear consensus that researchers were expected to get specific consent where practical and would have to explicitly justify to the HDEC any proposed non-consensual research use of tissue or data.

> You know, the first thing is; get consent. [HDEC4]

> I suppose the first question is; is it possible to get consent at all? And if so, how much of a problem would that be? [HDEC1]

There was some discussion of presumed or opt-out consent, and for the most part participants were skeptical of the moral work that presumed consent could do (note that presumed consent is not accepted as a valid authorization method under the NZ NEAC research ethics guidelines).

> You can't then assume that everybody would say "yes"; it's like having presumed consent, and that's not consent at all. [HDEC1]

In the absence of consent, participants wanted to know how much the proposed research deviated from the original consent and considered the degree to which the proposed use would be consistent with the *expectations* of the patient.

> What sort of consent was provided by those persons who provided that tissue in the first place? … What was the basis upon which that tissue was procured … For me, I think that's one of the more critical questions. [I2]

There was consensus that "practical barriers" were the strongest justification for not getting patient consent, and this included where patients were deceased

or the number of participants was too large. Ensuring the scientific validity of the study was also recognized as a compelling justification for research without consent. For nearly all participants, the cost of seeking consent was not considered adequate justification for failing to get consent. A minority of participants (especially those with a background in interventional research) disagreed and thought that cost was a relevant consideration in determining the practicality of getting consent.

Public benefit

As per the legislation and guidelines cited in the preceding, RECs in New Zealand can waive the patient consent requirement when the research is in the public interest. Other terms that were used by participants to refer to the idea of public interest include *public good*, *common good*, and *social value*. In the absence of a definition of *public good* in the national guidelines, participants struggled to develop criteria for assessing the social value of the proposed research.

> I mean doing this actually makes you really realize how ill-equipped we are to be objective … We're in a position where you've got to try and think what people, who've not been asked a question, might actually say to it. And the answer is, they'll all say something different. [HDEC2]

Participants' comments demonstrate some ambiguity regarding how to measure the benefit of data or tissue studies. At some points, discussion focused on whether the proposed research would generate new knowledge that would enrich our understanding of health and disease: "Is that actual technique you use going to be of sufficient, additional benefit to what we already know?" [I1].

However, a lot of the discussion, particularly in relation to data research, focused on whether the knowledge generated by the research would be used to promote the public good. Here the emphasis was on how the knowledge would be operationalized or implemented, rather than on whether the research would contribute to knowledge. Evaluating the potential future uses of knowledge led participants to consider political agendas, the broader policy social environment, and potential negative externalities arising from the research, such as loss of public trust.

> After all you don't know what the social benefit is, because it's research, and you don't know what the outcome's going to be. [HDEC1]

> When you start to collect, in this case, data on criminal activity, or data on groups that are outside the mainstream in their behavior, albeit not illegal, there really is the potential for, you know, for political interference. [HDEC2]

> How will doing this study increase access for Māori [the New Zealand indigenous population], because it doesn't seem like it will at all. I mean … the information doesn't help you get more people into a [health program]. [HDEC4]

The participants expressed significant suspicion of the increasing demand for health data, especially for large linking projects or big data analytics.

> Wanting to do all this grand linking. It's a bit like *Nineteen Eighty-Four*[2], isn't it? [HDEC3]

> I think I'm quite suspicious actually. And this committee has taught me to be more suspicious. Because the one thing I've learnt … is that the minute you open the can of worms, there really are worms in there. [HDEC2]

> The idealist in me would like to say no to the whole lot of them. [HDEC1]

Potential harms

Three core risks dominated discussion of potential harms: (1) stigmatization, and the potential to solidify stereotypes about marginalized populations; (2) loss of trust in clinicians and social service providers who originally collected the tissue or data; and (3) potential privacy harms. Other potential harms that participants mentioned included population surveillance, harms of predictive risk modeling, and withholding results of genetic/genomic research from patients or families.

> And also the surveillance, big brother stuff, particularly around youth crime and some of those kind of variables. I mean I think there would be lots of public trust concerns about that. [HDEC 3]

Potential threats to trust in particular arose often in discussion. The role of trust in various relationships was discussed—public trust in RECs/IRBs, RECs/IRBs' trust in researchers, patients' trust in clinicians, and communities' trust in the government.

> This research may have a great deal of social benefit, but … If it has a destructive effect on trust, [you get] a short-term benefit for long-term loss … we always need to think about that, because it's important that people trust their clinicians. [HDEC 1]

Standards

During the discussion, participants used different lenses for determining whether the proposed research

[2]This is a reference to George Orwell's dystopian novel about omnipresent government surveillance.

would be ethically appropriate. Three potential standards were used—the actual public, a reference population, and a hypothetical reasonable person. In some cases participants referred to their experience with the members of the general public or their knowledge of public surveys relating to secondary use research. The general view expressed was that most members of the public neither know nor care about secondary research with their data or tissue.

> Because one of our other members will say; she consents people all the time, she'll say; nobody ever cares about this. But it's our job to care about it for the one person that might. [HDEC4]

In other cases, participants referred to reference populations to try to determine whether the research was acceptable. This was presented as a more manageable task than determining what the public generally might think. Reference populations that were used in the discussion were Māori, Pasifika peoples,[3] young people, parents, and specific patient groups. For example, in the following, the participant references the views of women undergoing cervical screening in relation to a data linkage project.

> I did a piece of work around re-collecting ethnicity data for women's cervical screening … and 95% of the women said; well why is that different in different places, this is stupid, do your systems not talk to each other? [HDEC3]

In the next quote, the participant is considering the impact of the unconsented tissue research for Māori.

> Well, there's the dignitary harm of things being done to tissue, particularly for Māori … particularly sending samples overseas, but also genetic testing in general. [HDEC3]

Participants noted that researchers are beginning to submit patient or community surveys to the REC in support of their application for a consent waiver. Participants thought these were generally useful but not determinative in establishing whether the research was indeed ethically acceptable.

> So I think that kind of thing is useful, but you know, taking one or a small group as a representation of a population is tricky. [HDEC3]

Finally, a hypothetical reasonable person standard was also used to determine whether it would be necessary to gain additional consent from patients for secondary use of their tissue or data.

> But I do find myself … asking myself "what would a reasonable person ask about, think about this" often? Is this the kind of information that a reasonable person would expect to have? [HDEC2]

Responsibility

Participants felt the weight of responsibility of trying to balance the competing interests of the social value of the research and interests of collectives and individuals. Discussion of responsibility was closely associated with the theme of trust presented in the preceding:

> I think it's a really, really difficult dilemma … because you do not want to stop research, and you also don't want to end up approving stuff that's ethically not right. [I1]

> It's quite a responsibility too, cause you're approving this on behalf of the public, do the public want this? … I find them harder to approve [than standard observational or interventional studies]. [HDEC3]

Participants felt that when patient consent is sought, the responsibility of determining whether the research protocol is acceptable is shared between the REC and the patient. But when approving waivers of consent, the responsibility rests solely with the REC and amounts to a considerable burden.

> You know, because I think we have an enormous responsibility and if we can sort of share the responsibility with the participant … If they're happy with it, I'm happy with it. But here we carry all of the responsibility. [HDEC1]

> Consent takes those [multiple competing] voices away, because it's not my choice anymore, but without consent, I feel I'm definitely losing sleep. [HDEC2]

This perceived responsibility resulted in anxiety and stress for many participants.

> It just leaves a bad taste in your mouth. [HDEC1]

> It caused my hairs to rise up on end. [HDEC1]

> Yes, I feel uncomfortable. [HDEC3]

Participants linked this feeling of discomfort to the lack of guidance provided to RECs to assist them in evaluating the ethical issues identified and also noted that waiver of consent applications were harder to complete within the allocated 25 minutes.

> We're sort of, operating in a little bit of the void … Or even "these are the issues to consider," I mean that's not in our guidance anywhere. [HDEC3]

This anxiety was expressed by three of the four committees. The fourth committee was unique in two respects—its members did not express a high degree

[3]Descendants of the Polynesian nations of the Cook Islands, Tonga, Niue, Samoa, Tuvalu, and Tokelau who live in New Zealand.

of anxiety in relation to waiver of consent applications, and they reordered their schedule on the day (including calling researchers and rescheduling their appointment times) in order to allow sufficient time to address the more complex applications. They would often return to the same application at various points throughout the meeting.

> We will talk for an hour on an application if we have to … I don't think we ever run out of time, ever, on an application that we are uncomfortable [with]. [HDEC4]

Discussion

For the most part, participants identified the core ethical issues that receive attention in the literature—justifications for waiving consent, public good, public trust, cultural issues, privacy, and surveillance. Much of this content is not articulated in the current version of the New Zealand research ethics guidelines (Ballantyne and Style 2017), suggesting that committee members were informed by public and academic discourse on these issues.

The literature suggests the public is more supportive of secondary research when it produces public benefit, and more reluctant to share tissue or data with commercial companies (Trinidad et al. 2012; Ipsos MORI 2014). Many of the participants' comments highlight an ambiguity regarding how to define the benefits and risks of data research. In particular, this was visible in consideration of what would count as public benefit—is it that the knowledge generated from the study would be relevant and reliable, or that the knowledge would be used wisely and its application to policy would produce increased utility for members of the public?

Assessment of public benefit in a traditional clinical research trial is limited to evaluating whether the study would make a valuable contribution to knowledge. The issue of whether funding agencies would be likely to subsequently fund a drug, if successful, is typically outside the scope of REC/IRB review. But in this study, HDEC members often attempted to judge research according to its anticipated future impact on the health system, rather than its contribution to knowledge.

Participants used three potential standards for assessing public benefit—the actual public, a reference population, and a hypothetical reasonable person. Participants noted limitations involved with each of these approaches and that different lenses would lead to different conclusions. Strategies for improving patient and public involvement (PPI) in research are gaining increasing attention in the literature (Sacristán et al. 2016) and seem especially relevant for nonconsensual studies. RECs/IRBs need a clear strategy on how to deal with evidence of patient perspectives. Participants in our study noted that researchers were increasingly submitting evidence of patient support for the research, and questioned what moral weight the committee should give this evidence. Patient feedback is clearly not equivalent to individual consent, and participants appeared to struggle to know how to assess this evidence. An explicit framework might help to avoid tokenistic initiatives (Domecq et al. 2014) to involve patients, encourage researchers to systematically and meaningfully engage with patients and data subjects, and help ensure nonconsensual research is in line with public expectations.

When adopting these different lenses, participants appeared to be trying to determine whether there was plausible hypothetical acceptance of the research—would patients likely consent to the study if asked? This is one way of approaching their task of determining that the public interest in the study outweighs the public interest in privacy (NEAC 2012). As such, participants appear to be adopting a "social license" perspective rather than an "ethical" perspective. Social license concerns whether the study is or would be acceptable to the public, while ethics concerns whether the study is correct or justifiable according to ethical principles. Sometimes there is a difference between what is ethical and what is socially acceptable (e.g., many would argue that slavery has always been ethically objectionable, even though it was socially acceptable for long periods of human history). It is not clear whether the role of RECs/IRBs should be to determine the ethical acceptability of research or whether there is social license for the research, or both, and what they should prioritize if these happen to diverge (Moore and Donnelly 2015).

The degree of distress and anxiety expressed by participants was striking and unexpected. Participants were not directly asked whether reviewing secondary use applications generated stress, but many of their comments indicated a high level of stress. In the absence of clear research ethics guidelines, committees are in limbo, having to generate rules on the fly and trying to ensure consistency across committees and cases. Other studies show that applying clinical research ethics guidelines to tissue and data research presents additional challenges for RECs/IRBs (Rothstein 2002; Wolf et al. 2008; Goldenberg et al. 2015). Participants' comments suggest that the stress was linked to uncertainty—lack of guidance,

uncertainty regarding how the research results will be put into practice, and uncertainty about whether the public or patients would endorse the research.

Interestingly, one of the four committees reported little anxiety about secondary use applications. This was also the committee that rearranged its agenda to provide more time for difficult applications. This suggests that extra time to consider secondary use research could partially compensate for the lack of explicit guidance.

By definition, nonconsented use of tissue and data places more responsibility with RECs/IRBs and other governance bodies such as Data Access Committees, simply because patient consent is not sought. But our research suggests that the weight of responsibility could be mitigated through clearer national guidance (especially where it reflects public consultation and engagement), sufficient time to work through the complex interests at stake, and clearer guidance on how to integrate evidence of PPI in their deliberations.

Many jurisdictions (e.g., New Zealand, Australia, and the United States) waive consent requirements if researchers can show that gaining consent is impractical. Can financial cost itself be a legitimate form of impracticality? The NZ NEAC Guidelines state the condition for waiver of consent as being that consent would be "impossible in practice" (NEAC 2012, s6.43). The recently revised Australian National Statement on Ethical Conduct in Human Research gives the following examples of "impracticable": due to the quantity, age, or accessibility of records (National Health and Medical Research Council 2015). A case study of the U.S. Cardiovascular Register cites financial cost and methodological reasons as justification for not gaining patient consent to use health data (Haynes, Cook, and Jones 2007). By contrast, many of the NZ HDEC members did not consider financial burden alone a sufficient justification for not seeking patient consent. Whether or not financial cost qualifies as a legitimate reason for waiving consent will have significant practical implications for secondary research.

Limitations

A limitation of the study is that we only focused on the experiences of the national-level HDECs and excluded institutional RECs and the one (at the time of the research) private New Zealand REC. Given that the HDECs have joint secretarial support provided by the MOH, they are likely to have greater consistency across the committees. We could expect to see more diversity of approach to risk assessment, guideline interpretation, and review among the institutional and private RECs.

A second limitation was the short time for the focus groups, given the complexity of the subject matter. This was necessary due to the general demands on committee members who were traveling to attend the scheduled meeting. We were able to compensate for this limitation, in part, with the follow-up interviews and the iterative engagement with the HDEC Chairs and MOH secretariat during the data analysis and interpretation process.

Conclusion

These findings have highlighted a number of important issues for stakeholders in REC/IRB review of secondary research. RECs/IRBs require detailed guidance regarding the processes and grounds for assessing secondary research use of tissue and data, in particular how to define and measure the social benefits of secondary research. RECs/IRBs would also benefit from explicit policy on the relevance of evidence presented by researchers of patient and public involvement (PPI). Guidelines should clarify whether cost is a sufficient ground for not seeking consent for secondary research (e.g., by being, in some circumstances, a form of impracticality). Finally, RECs/IRBs may require more time to consider nonconsented studies, as these are complex and place a higher burden of responsibility on the committees.

Conflicts of interest

The authors have no conflicts of interest to declare.

Acknowledgments

We would like to thank the participants and the Ministry of Health Secretariat for the Health and Disability Ethics Committees for their time and assistance; and Wendy Rogers, Agomoni Ganguli-Mitra, and Maria Stubbe for helpful advice and feedback.

Author contributions

Angela Ballantyne and Andrew Moore conceived of the study; AB applied for ethics approval, was responsible for recruitment, data collection and coding; AB and AM were jointly responsible for data interpretation, analysis and drafting the paper.

Funding

The project was funded by the Royal Society New Zealand (Marsden Fund UOO1515).

References

Ballantyne, A., and R. Style. 2017. Health data research in New Zealand: Updating the ethical governance framework. *The New Zealand Medical Journal* 130(1464): 64–71.

Borgerson, K. 2014. Redundant, secretive, and isolated: when are clinical trials scientifically valid? *Kennedy Institute of Ethics Journal* 24(4):385–411.

Caplan, A. L. 1984. Inconsistency, idiosyncrasy, and IRBs. *International De Recherches Betteravieres* 6(2):10

Council for International Organizations of Medical Sciences (CIOMS). 2016. International Ethical Guidelines for Health-related Research Involving Humans, Fourth Edition. Geneva: Council for International Organizations of Medical Sciences. http://cioms.ch/ethical-guidelines-2016/WEB-CIOMS-EthicalGuidelines.pdf.

Davidson, S., C. McLean, S. Treanor., et al. 2013. Public acceptability of data sharing between the public, private and third sectors for research purposes. Scottish Government Social Research. http://www.scotland.gov.uk/socialresearch.

Domecq, J. P., G. Prutsky, T. Elraiyah, Z. Wang, M. Nabhan, N. Shippee, J. P. Brito, K. Boehmer, R. Hasan, B. Firwana., et al. 2014. Patient engagement in research: A systematic review. *BMC Health Services Research* 14:89.

Goldenberg, A. J., K. J. Maschke, S. Joffe, J. R. Botkin, E. Rothwell, T. H. Murray, R. Anderson, N. Deming, B. F. Rosenthal, S. M. Rivera., et al. 2015. IRB practices and policies regarding the secondary research use of biospecimens. *BMC Medical Ethics* 16(1):32. doi:10.1186/s12910-015-0020-1.

Goldman, J., and M. D. Katz. 1982. Inconsistency and institutional review boards. *JAMA* 248(2):197–202.

Grant, A., J. Ure, D. J. Nicolson, J. Hanley, A. Sheikh, B. McKinstry, and F. Sullivan. 2013. Acceptability and perceived barriers and facilitators to creating a national research register to enable 'direct to patient' enrolment into research: the scottish health research register (SHARE). *BMC Health Services Research* 13(1):422. doi:10.1186/1472-6963-13-422.

Hammersley, M. 2010. Creeping ethical regulation and the strangling of research. *Sociological Research Online* 15(4): 1. http://www.socresonline.org.uk/15/4/16.html.

Haynes, C. L., G. A. Cook, and M. A. Jones. 2007. Legal and ethical considerations in processing patient-identifiable data without patient consent: lessons learnt from developing a disease register. *Journal of Medical Ethics* 33(5):302–7. doi:10.1136/jme.2006.016907.

HDC Code of Health and Disability Services Consumers' Rights Regulation. 1996. (NZ). http://www.hdc.org.nz/the-act–code/the-code-of-rights/the-code-(full).

Health Information Privacy Code 1994. (NZ) https://www.privacy.org.nz/the-privacy-act-and-codes/codes-of-practice/health-information-privacy-code-1994/.

Human Tissue Act 2008. (NZ) http://www.legislation.govt.nz/act/public/2008/0028/latest/DLM1152940.html.

Ipsos MORI 2014. Dialogue on Data. https://www.ipsos.com/ipsos-mori/en-uk/dialogue-data.

Kawulich, B. 2005. Participant observation as a data collection method. *Qualitative Social Research* 6(2). http://www.qualitative-research.net/index.php/fqs/article/view/466/996.

Kaye, J., E. A. Whitley, D. Lund, M. Morrison, H. Teare, and K. Melham. 2015. Dynamic consent: a patient interface for twenty-first century research networks. *European Journal of Human Genetics* 23(2):141–6. doi:10.1038/ejhg.2014.71.

Klitzman, R. 2013. How IRBs view and make decisions about coercion and undue influence. *Journal of Medical Ethics* 39(4):224–9. doi:10.1136/medethics-2011-100439.

Menikoff, J., J. Kaneshiro, and I. Pritchard. 2017. The common rule, updated. *New England Journal of Medicine* 376(7):613–5. 16. doi:10.1056/NEJMp1700736.

Ministry of Health (NZ) 2014. *Standard operating procedures for health and disability ethics committees*. Wellington: Ministry of Health. http://ethics.health.govt.nz/operating-procedures.

Moore, A., and A. Donnelly. 2018. The job of 'ethics committees'. *Journal of Medical Ethics* 44:481–487.

National Ethics Advisory Committee (NZ) 2012. *Ethical guidelines for observational studies: Observational research, audits and related activities*. Revised edition. Wellington: Ministry of Health.

National Health and Medical Research Council (NHMRC) 2015. Chapter 2.3: Qualifying or waiving conditions for consent. National Statement on Ethical Conduct in Human Research (2007) (Updated May 2015). https://www.nhmrc.gov.au/book/national-statement-ethical-conduct-human-research.

Ploug, T., and S. Holm. 2016. Meta Consent - A flexible solution to the problem of secondary use of health data. *Bioethics* 30(9):721–32. doi:10.1111/bioe.12286.

Privacy Act. 1993. (NZ). http://www.legislation.govt.nz/act/public/1993/0028/latest/DLM296639.html.

Rothstein, M. A. 2002. The role of IRBs in research involving commercial biobanks. *The Journal of Law, Medicine & Ethics* 30(1):105–8. doi:10.1111/j.1748-720X.2002.tb00726.x.

Sacristán, J. A., A. Aguarón, C. Avendaño-Solá, P. Garrido, J. Carrión, A. Gutiérrez, and A. Flores. 2016. Patient involvement in clinical research: why, when, and how. *Patient Preference and Adherence* 10 :631–40. doi:10.2147/PPA.S104259

Smith, M., F. Doyle, H. M. McGee, and D. De La Harpe. 2004. Ethical approval for national studies in Ireland: an illustration of current challenges. *Irish Journal of Medical Science* 173(2):72.

Stark, L. 2012. *Behind closed doors: IRBs and the making of ethical research*. Chicago: University of Chicago Press.

Trace, S., and S. E. Kolstoe. 2017. Measuring inconsistency in research ethics committee review. *BMC Medical Ethics* 18(1):65. doi:10.1186/s12910-017-0224-7.

Transparency International. 2017. http://www.transparency.org.nz/.

Trinidad, S. B., S. M. Fullerton, J. M. Bares, G. P. Jarvik, E. B. Larson, and W. Burke. 2012. Informed consent in

Genome-Scale research: What do prospective participants think? *AJOB Prime Research* 3(3):3–11.

Wenner, D. M. 2016. Barriers to effective deliberation in clinical research oversight. *HEC Forum* 28(3):245–59. doi: 10.1007/s10730-015-9298-0.

Willison, D. J., K. Keshavjee, K. Nair, C. Goldsmith, and A. M. Holbrook. 2003. Computerization of Medical Practices for the Enhancement of Therapeutic Effectiveness investigators. Patients' consent preferences for research uses of information in electronic medical records: interview and survey data. *BMJ* 326(7385):373.

Wolf, L. E., J. A. Catania, M. M. Dolcini, L. M. Pollack, and B. Lo. 2008. IRB chairs' perspectives on genomics research involving stored biological materials: ethical concerns and proposed solutions. *Journal Empire Research Human Research Ethics* 3(4):99–111. doi:10.1525/jer.2008.3.4.99.

World Medical Association. 2016. Declaration of Taipei on Ethical Considerations Regarding Health Databases and Biobanks. https://www.wma.net/policies-post/wma-declaration-of-taipei-on-ethical-considerations-regarding-health-databases-and-biobanks/.

Xafis, V. 2015. The acceptability of conducting data linkage research without obtaining consent: lay people's views and justifications. *BMC Medical Ethics* 16(1):79. doi: 10.1186/s12910-015-0070-4.
